# Traumatic Brain Injury in the Elderly: Clinical Features, Prognostic Factors, and Outcomes of 133 Consecutive Surgical Patients

**DOI:** 10.7759/cureus.13587

**Published:** 2021-02-27

**Authors:** Guilherme G Podolsky-Gondim, Rodrigo Cardoso, Edson Luis Zucoloto Junior, Luca Grisi, Mateus Medeiros, Stephanie Naomi De Souza, Marcelo V Santos, Benedicto O Colli

**Affiliations:** 1 Division of Neurosurgery, Department of Surgery and Anatomy, Ribeirão Preto Clinics Hospital, Ribeirão Preto Medical School, University of São Paulo, Ribeirão Preto, BRA; 2 Division of Pediatric Neurosurgery, Department of Surgery and Anatomy, Ribeirão Preto Clinics Hospital, Ribeirão Preto Medical School, University of São Paulo, Ribeirão Preto, BRA

**Keywords:** elderly, geriatric trauma, head injury, subdural hematoma, traumatic brain injury, anticoagulants

## Abstract

Objective

With the aging of the global population, an increase in the proportion of elderly patients presenting with traumatic brain injury (TBI) is expected. This population presents several distinctive characteristics that impact management and outcome of TBI, such as comorbidities, frailty, and preinjury use of medications - specially antiplatelets and anticoagulants. The purpose of this study was to assess the general characteristics and prognostic factors of elderly patients with TBI that were surgically managed at a single institution.

Methods

The authors performed a retrospective review of all elderly patients (age ≥ 65 years) with a history of TBI that underwent cranial neurosurgical procedures at their institution, between 2015 and 2019. Clinical characteristics, laboratory tests, and radiological scans, as well as surgeries, performed, outcome, and prognostic factors were analyzed, comprising 133 consecutive cases overall.

Results

The mean age of patients was 76.6 ± 7.3 years, ranging from 65 years to 97 years. There was a predominance of males (71.4%) and the most frequent mechanism of injury was fall (80.4%). Mild TBI comprised 57.1% of the cases, followed by severe TBI in 25.6%. Frequent signs and symptoms were impaired consciousness (69.9%), focal motor deficits (32.3%), and gait disturbances (12.8%). The majority had reported comorbidities upon admission (79.7%), with cardiac disease (79.2%) and diabetes (24.5%) as the most frequent. Preinjury anticoagulation was reported in 18.8% and use of antiplatelet drugs in 17.3%. The most common finding in the head CT was chronic subdural hematoma (48.1%), followed by acute subdural hematoma (37.6%). Coagulation was found to be altered in 12.8% of the patients. The most common neurosurgical procedure performed was trephination for hematoma evacuation (56.3%), followed by craniotomy (21.2%). Blood product transfusion was needed in 61.7% of the patients. Overall mortality was 42.1%, with the majority in the first month after admission (83.9%). Unfavorable outcome (Glasgow Outcome Scale <5) at discharge was identified in 73% of the patients. Identified prognostic factors were TBI severity, absent pupillary reactivity, acute intracranial bleeding on head CT, basal cisterns obliteration, altered coagulation status, and need for blood transfusion.

Conclusions

TBI severity, pupillary reactivity, coagulation status, need for blood products transfusion and acute bleeding, as well as basal cisterns obliteration found in head CT, are factors that influenced the outcome in this series of elderly patients with TBI that need surgical management. It is paramount to observe the particularities of this population in this context, to optimize outcomes, avoid complications and ultimately generate awareness focused on prevention.

## Introduction

It is currently well-known that the global population is aging, with estimates from the United Nations that one in six people in the world will be over 65 years by 2050 [1]. In the United States, it is expected that by 2034, the population older than 65 years will overpass the population under 18 years [2]. Likewise, in Brazil, it is estimated that 25% of the population will be over 65 years by 2060 - currently, this proportion is 9.2% [3].

Traumatic brain injury (TBI) has three peak incidences, occurring mainly among children, young adults, and the elderly [4]. TBI alone results in more than 80,000 emergency department visits per year in patients aged 65 years or older in the United States. Out of this number, approximately 75% lead to patient hospitalization. And the leading causes of TBI in this population were falls (51%) followed by motor vehicle collisions (9%) and assaults (1%) [5].

Unlike their younger counterparts, the most common TBI manifestations in the elderly are contusions and subdural and intracerebral hemorrhage. The physiological changes that occur in this population explain the differences in the patterns of brain injury - reduced brain volume associated with increased subdural space, fragile walls of cerebral vessels - especially bridging veins - and adherence of the dura-mater to the inner surface of the cranium [6]. In addition, preexisting chronic medical conditions, such as diabetes, heart and kidney diseases, negatively impact survival in this population [7]. Also, preinjury use of anticoagulant and antiplatelet drugs poses a challenge for the management at the emergency department, dramatically increasing morbidity and mortality in the elderly [8-12].

The goal of this study was to analyze the clinical characteristics and prognostic factors of a consecutive series of elderly patients that underwent neurosurgical procedures at our institution following TBI.

## Materials and methods

Population studied

The authors performed a retrospective review of records and radiological scans of all elderly patients (age ≥ 65 years upon admission) with a diagnosis of TBI and who underwent any TBI-related neurosurgical procedure at the University Hospital of Ribeirão Preto Medical School, São Paulo, Brazil, a level I regional trauma center, between 2015 and 2019. Patients with radiological findings of chronic subdural hematoma, but no confirmed history of trauma were excluded from this study.

All acute trauma patients were evaluated according to the Advanced Trauma Life Support guidelines of the American College of Surgeons, and further radiological and neurosurgical assessments were employed whenever deemed necessary by the admitting team. The remaining patients, for which there were no acute injuries (55% of total), were admitted to the neurosurgical ward for observation.

Data were obtained from electronic medical charts and included demographic information, mechanism of injury, known comorbidities, status of anticoagulation and platelet antiaggregation, prescription drugs, clinical signs, and symptoms upon admission - including pupillary reaction - associated lesions, head computed tomography findings, laboratory results, surgeries performed, length of stay, complications, need for blood transfusion, outcomes (as measured by the Glasgow Outcome Scale - GOS - at discharge, 30 days, 60 days, six months, and one year after admission, if available) and mortality. Outcome was classified in favorable (GOS = 5) and unfavorable (GOS <5).

The serum lactate and base deficit levels on admission were categorized as described by Callaway et al., for their known role as prognostic biomarkers; the lactate level was classified as normal (0 to 2.4 mmol/L), moderately elevated (2.5 to 4.0 mmol/L), or severely elevated (>4.0 mmol/L) and base deficit was classified as normal (> 0 mEq/L), moderate (0 to -6 mEq/L) or severe (< -6 mEq/L) [13]. Coagulation status was assessed by fibrinogen level and prothrombin time - expressed by international normalized ratio (INR) and classified as non-therapeutic (<2.0) or altered (≥ 2.0).

Institutional approval for this study was obtained from the Ethics Committee of the University Hospital of Ribeirão Preto Medical School - University of São Paulo.

Statistical analysis

GraphPad Prism 8 (version 8.4.2, GraphPad Software, San Diego, CA) was used for the statistical analyses. Descriptive data were presented as means (with standard deviation), median - when applicable, and proportions. Univariate analysis was performed with Fisher’s exact test for categorical variables, except for admission Glasgow Coma Scale, lactate level and base deficit, which were analyzed with chi-square. Odds-ratio (OR) with 95% confidence intervals (CI) were calculated. Statistical significance was assumed when ɑ = 0.05.

## Results

A total of 133 elderly patients who matched the including criteria were identified. Table 1 summarizes their clinical characteristics. Most patients were male (95/71.4%). The mean age was 76.6 ± 7.3 years (range 65-97 years). The predominant mechanism of trauma was fall (107/80,4%), followed by traffic accident - pedestrian (9/6.8%), traffic accident - vehicle occupant (6/4.5%), and assault (2/1.5%). Mean Glasgow Coma Scale (GCS) on admission was 11.3 ± 3.9, with most patients presenting with mild TBI - GCS 13 to 15 - (76/57.1%), followed by severe TBI - GCS <9 - (34/25.6%) and moderate - GCS 9-12 - (23/17.3%). Frequent signs and symptoms upon presentation were impaired consciousness (93/69.9%), focal motor deficits (32.3%), gait disturbances (17/12.8%), and speech impairment (16/12%). Pupils were equal and reactive in most patients (110/82.7%), followed by unequal pupils (20/15%) and non-reactive (3/2.3%). Comorbidities were reported in approximately 80% of the patients, with cardiac disease, mainly chronic arterial hypertension and cardiac failure, being the most frequent (84/79.2%), followed by diabetes (26/24.5%) and chronic neurologic conditions, such as dementia or epilepsy (26/24.5%). Chronic alcohol use disorder was reported in 6.6% of the patients. Use of anticoagulant drugs prior to admission was reported in 25 patients (18.8%), warfarin being the most frequently used one (20/80%), followed by rivaroxaban (3/12%) and dabigatran (1/4%). Use of platelet antiaggregant agents was reported in 17.3% of patients, with aspirin being the more frequent one (22/95.6%), followed by clopidogrel (4/17.4%) and cilostazol (1/4.3%).

**Table 1:** Clinical characteristics of 133 geriatric patients with surgical head trauma SD: standard deviation; GCS: Glasgow Coma Scale

Characteristic	Value
Age (years)	
Range	65-97
Mean age ± SD	76.6 ± 7.3
Median age	76
Sex	
Male	95 (71.4%)
Female	38 (28.6%)
Mechanism of injury	
Fall	107 (80.4%)
Traffic accident (pedestrian)	9 (6.8%)
Traffic accident (vehicle occupant)	6 (4.5%)
Assault	2 (1.5%)
Not specified	9 (6.8%)
Severity	
GCS 13-15	76 (57.1%)
GCS 9-12	23 (17.3%)
GCS 3-8	34 (25.6%)
Mean GCS ± SD	11.3 ± 3.9
Median GCS	13
Signs and symptoms	
Impaired consciousness	93 (69.9%)
Focal motor deficits	43 (32.3%)
Gait disturbance	17 (12.8%)
Speech disturbances	16 (12%)
Headache	11 (8.3%)
Seizures	7 (5.3%)
Vomiting	4 (3%)
Visual loss	2 (1.5%)
Urinary incontinence	2 (1.5%)
Focal sensory deficit	1 (<1%)
Dizziness	1 (<1%)
Asymptomatic	1 (<1%)
Pupillary reactivity	
Equal/reactive	110 (82.7%)
Unequal	20 (15%)
Non-reactive	3 (2.3%)
Comorbidities	
No/unknown	27 (20.3%)
Yes	106 (79.7%)
Cardiac	84 (79.2%)
Diabetes	26 (24.5%)
Neurologic	26 (24.5%)
Arrhythmia	11 (10.4%)
Other endocrinological pathology	11 (10.4%)
Respiratory	10 (9.4%)
Renal	10 (9.4%)
Vascular	7 (6.6%)
Oncological	8 (7.5%)
Alcohol abuse	7 (6.6%)
Other	31 (29.2%)
Anticoagulation status previous to admission	
No/unknown	108 (81.2%)
Yes	25 (18.8%)
Warfarin	20 (80%)
Rivaroxaban	3 (12%)
Dabigatran	1 (4%)
Not specified	1 (4%)
Use of platelet antiaggregants	
No/unknown	110 (82.7%)
Yes	23 (17.3%)
Aspirin	22 (95.6%)
Clopidogrel	4 (17.4%)
Cilostazol	1 (4.3%)

Table 2 summarizes the radiological and laboratory findings. The most common head CT findings were chronic subdural hemorrhage (64/48.1%), acute subdural hemorrhage (50/37.6%), and intracerebral hemorrhage (11.3%). Approximately 57% of the patients had a midline shift equal to or greater than 5 mm, but only 15% of the patients had signs of basal cisterns obliteration on their initial CT. Mean hemoglobin level was 12.7 ± 2.3 mg/dL and mean platelet level was 210.2 ± 78.4 100x10^3^/mm^3^. Upon admission, approximately fifty percent of patients (63 patients) had normal serum lactate levels and 63% had moderate base deficit (62 patients). Coagulation status was measured by prothrombin time and presented as per the INR, with a mean of 1.59 ± 1.7 (range 0.86-11.4) and was considered altered (INR ≥ 2.0) in approximately 12.8% of patients, including two patients with an uncoagulable status. Fibrinogen was measured in thirty patients and was considered reduced (< 180 mg/dL) in five patients (16.7%).

**Table 2:** Radiological and laboratory findings in 133 geriatric patients with surgical head trauma SD: standard deviation; INR: international normalized ratio

Exam and Findings	
Head computed tomography	
Injuries	
Chronic subdural hematoma	64 (48.1%)
Acute subdural hematoma	50 (37.6%)
Intracerebral hemorrhage	15 (11.3%)
Cerebral contusion	10 (7.5%)
Traumatic subarachnoid hemorrhage	10 (7.5%)
Hygroma	2 (1.5%)
Epidural hematoma	1 (0.75%)
Midline shift	
< 5 mm	57 (42.9%)
≥ 5 mm	76 (57.1%)
Basal cisterns obliteration	
No	113 (85%)
Yes	20 (15%)
Laboratory results	
Hemoglobin (mg/dL)	
≥ 8.0	128 (96.2%)
< 8.0	5 (3.8%)
Mean ± SD	12.7 ± 2.3
Median	13
Platelets (x 10^3^/mm^3^)	
≥ 100	8 (6%)
< 100	125 (94%)
Mean ± SD	210.2 ± 78.4
Median	205
Serum lactate (mmol/L)	
0-2.4 mmol/L (normal)	32 (50.8%)
2.5-4.0 mmol/L (moderately elevated)	17 (27.0%)
> 4.0 mmol/L (severely elevated)	14 (22.2%)
Mean ± SD	3.24 ± 2.65
Median	2.3
Base lactate deficit (mEq/L)	
> 0 mEq/L (normal)	9 (14.5%)
0 to -6 mEq/L (moderate)	39 (62.9%)
< -6 mEq/L (severe)	14 (22.6%)
Mean ± SD	-3.53 ± 3.39
Median	-3.05
Prothrombin time (INR)	
Not therapeutic (< 2.0)	116 (87.2%)
Altered (≥ 2.0)	17 (12.8%)
Uncoagulable	2 (1.5%)
Mean ± SD	1.59 ± 1.7
Median	1.09
Fibrinogen (mg/dL)	
≥ 180 mg/dL	25 (83.3%)
< 180 mg/dL	5 (16.7%)
Mean ± SD	271.2 ± 121
Median	265.5

A total of 151 surgical procedures were performed for this series of patients. Table 3 reports the main findings regarding the type of surgery, complications, tracheostomy, and blood transfusion. The most common procedures were trephination for subdural hematoma evacuation (85/56.3%), craniotomy (32/21.2%), and decompressive craniotomy (17/11.25%). Five patients (3.3%) were reoperated, either by craniotomy or reopening of the burr holes. Other complications recorded were related to the surgical wound - either dehiscence or infection (6/4.5%) - and thromboembolic events (5/3.75%), such as deep vein thrombosis or pulmonary thromboembolism.

**Table 3:** Surgical procedures and related features in 133 geriatric patients with surgical head trauma ICP: intracranial pressure; EVD: external ventricular drain; LD: lumbar drain

Surgical Procedure (Total = 151)	
Trephination(s)	85 (56.3%)
Craniotomy	32 (21.2%)
Decompressive hemicraniectomy	17 (11.3%)
ICP monitoring (exclusively)	4 (2.6%)
Debridement	3 (2%)
EVD/LD	3 (2%)
Reoperation (craniotomy or trephination)	5 (3.3%)
Other	2 (1.3%)
Complications	
Related to the surgical wound	6 (4.5%)
Deep vein thrombosis/pulmonary thromboembolism	5 (3.8%)
Tracheostomy	
No	107 (80.4%)
Yes	26 (19.6%)
Blood products transfusion	
No	82 (61.7%)
Yes	51 (38.3%)

Finally, clinical outcome and analysis of prognostic factors are reported in Table 4. Overall mortality was 42.1% (56 patients), with 83.9% reported in the first months after admission. Thirty-six patients (27%) had a favorable outcome upon discharge, with a reported decreased proportion in the next time periods (30 days, 60 days, six months, and one year after admission), secondary to loss of follow-up and discharge from the outpatient clinic. Prognostic factors that significantly affected overall mortality and GOS at discharge were TBI severity, pupillary reactivity (OR 9.372, CI 3.143-26.5), acute bleeding in head CT (OR 3.581, CI 1.598-7.519), obliteration of basal cisterns (OR 7.3, CI 2.349-20.9) and transfusion of blood products (OR 5.455, CI 2.498-11.14). Altered prothrombin time (INR ≥ 2) was found to affect overall mortality (OR 3.927, CI 1.254-10.51).

**Table 4:** Mortality, outcomes, and prognostic factors in 133 geriatric patients with surgical head trauma OR: odds ratio; CI: confidence interval; GOS: Glasgow Outcome Scale; GCS: Glasgow Coma Scale; INR: international normalized ratio (*) - Statistically significant / (N/A) – assumptions not fulfilled for the chi-square test.

Mortality				n (%)				
Overall				56 (42.1%)				
30 days				47 (35.3%)				
60 days				54 (39.9%)				
6 months				55 (41.4%)				
12 months				56 (42.1%)				
Clinical outcome	Discharge		30 days		6 months		1 year	
Favorable (GOS 5)	36 (27%)		29 (26.6%)		13 (16.25%)		11 (14.5%)	
Unfavorable (GOS <5)	97 (73%)		80 (73.4%)		67 (83.75%)		65 (85.5%)	
N (% of total)	133 (100%)		109 (81.9%)		80 (60.1%)		76 (57.1%)	
Prognostic factors		Mortality			Outcome at discharge			
		OR (95% CI)	p-Value		OR (95% CI)		p-Value	
Sex								
Male		1.358 (0.6399-2.870)	0.5602		0.7816 (0.3317-1.922)		0.6688	
Severity			<0.0001*				0.0009*	
Pupillary reactivity								
Unequal		9.372 (3.143-26.50)	<0.0001*		4.697 (1.227-21.06)		0.0373*	
Anticoagulation previous to admission								
Yes		2.451 (0.9913-6.103)	0.0707		1.610 (0.5966-4.198)		0.4604	
Previous use of platelet antiaggregant								
Yes		0.8617 (0.3645-2.089)	0.8194		1.949 (0.6458-5.608)		0.3100	
Type of CT bleeding								
Acute		3.581 (1.598-7.519)	0.0015*		2.813 (1.215-6.189)		0.0163*	
Midline shift								
≥ 5 mm		1 (0.5011-2.022)	>0.9999		0.7992 (0.3741-1.730)		0.6939	
Basal cisterns obliteration								
Yes		7.300 (2.349-20.90)	0.0003*		Infinity (2.418-Infinity)		0.0018*	
Platelets (x 103/mm3)								
< 100		1.404 (0.3930-4.990)	0.7205		2.722 (0.4555-31.46)		0.6823	
Serum lactate			0.3143				N/A	
Base lactate deficit			N/A				N/A	
Prothrombin time (INR)								
Altered (≥ 2.0)		3.927 (1.254-10.51)	0.0165*		1.855 (0.5556-6.371)		0.5591	
Fibrinogen								
< 180 mg/dL		2.250 (0.2686-30.24)	0.6400		Infinity (0.1410-Infinity)		>0.9999	
Blood products transfusion								
Yes		5.455 (2.498-11.14)	<0.0001*		5.592 (2.087-14.01)		0.0003*	

## Discussion

The main mechanisms of injury in this series were fall (80.4%), motor vehicle collision (11.3%) - pedestrian and vehicle occupant - and assault (1.5%), but no gun-related injuries. However, there was a predominance of fall over the remaining mechanisms, when comparing with other reported series (30-51% of trauma cases in this age group) that account for elderly patients with TBI in general - surgical and conservative cases [8,14,15]. This may be explained by previously described studies depicting the higher injury severity and increased need for surgical management of elderly patients that present with this type of mechanism [16]. 

Clinical presentation was variable, however, 69.9% of the patients had an impaired consciousness upon admission, followed by focal motor deficits and gait disturbances. This raises the importance to alert caretakers and family members for changes in behavior, focal weakness, and gait disturbances, signs that may be overlooked and mistakenly attributed to physiological consequences of aging, leading to underreporting and failure to seek medical attention promptly [17]. Also, an episode of fall may be unnoticed or the TBI ignored by caretakers or medical staff because of distracting lesions, such as orthopedic trauma [18].

A large study assessing the influence of preexisting medical conditions in elderly trauma patients identified 33% of patients with at least one comorbidity and it was associated with an increased risk of death [7]. In this study, approximately 80% of the patients had at least one reported chronic disease upon admission, thus suggesting a higher risk of surgically managed TBI in this population. Also, it is important to consider the prevalence of acute and chronic abusive alcohol drinking in this population. It is reported that in elderly patients 13% of men and 8% of women reported at-risk alcohol use, with respectively 14% and 3% of binge drinking [19]. In our series, chronic abusive alcohol use was reported in 6.6% of the patients.

The role of polypharmacy (i.e., the use of multiple medications and/or the administration of more medications than are clinically indicated, representing unnecessary drug use) spanning from inappropriate use of prescribed medicines, as well as self-medicating without an indication, in this age group is associated with the high prevalence of comorbidities [20].

Preinjury use of anticoagulant or antiplatelet drugs is an example of a worrisome issue in this population, due to the higher risk of complications and mortality following low energy traumas, such as falls. Besides the prompt identification of the coagulation status of the elderly patient with TBI, is the need for urgent reversal of anticoagulation or antiplatelet agents, when a TBI is suspected, in order to reduce the risk of mortality or unfavorable outcome [21-24]. Although 18.8% of the patients in this series were reported to use anticoagulation medicine previous to admission, only the coagulation status assessed by prothrombin time (≥2.0) was found to significantly affect mortality, thus reinforcing that anticoagulation per se doesn’t affect the outcome, but the anticoagulation status does [25].

The high mortality and predominant proportion of unfavorable outcomes in this series underscore the importance of the already reported specific protocols for the management of elderly patients with TBI. Protocols spanning from hospital admission, inpatient care - if possible, in a dedicated unit or early admission to an intensive care unit - and transfer to a rehabilitation facility or discharge with an integral follow-up [26-30]. Also, it is paramount to address the prevention of this type of trauma, by enrolling interventions focused both in the host/agent and the environment, in all the phases regarding the event - for example, in the pre-event phase addressing polypharmacy, improving strength and balance and eliminating environmental hazards [5].

Limitations

The major limitation of this study is that it only comprises elderly patients with TBI that underwent any neurosurgical procedure following admission. This limitation is due to the nature of the database used - the registry of all neurosurgical procedures of our institution. Thus, it is expected that the results will be impacted towards worse clinical presentations, higher mortality, and unfavorable outcomes. Also, patients that had a favorable outcome upon hospital discharged, were commonly discharged from the neurosurgery outpatient clinic if no complications or limitations were detected, thus reducing the data available for outcome measures at 60 days, six months, and one-year post-admission. Another limitation may be that not all the patients had been tested for fibrinogen, serum lactate, and base deficit levels upon admission - in our institution only the severe and acute trauma patients have the blood gases and fibrinogen tested, thus reducing the statistical power of the analysis of these factors.

## Conclusions

The ongoing transition in the epidemiology of TBI following the aging of the global population highlights the necessity of special attention to the peculiarities of this group, with an emphasis on the physiological changes, comorbidities, and use of multiple medications as factors that impact the outcome, obviating the need to raise awareness towards TBI prevention in this particular group. Factors that affect prognosis in the population studied herein are TBI severity, pupillary reactivity, bleeding type as found in head CT, basal cisterns obliteration, anticoagulation status, and transfusion of blood products.
